# A golden approach to ion channel inhibition^☆^

**DOI:** 10.1016/j.tips.2013.07.004

**Published:** 2013-09

**Authors:** Gavin E. Jarvis, Andrew J. Thompson

**Affiliations:** 1 Department of Physiology, Development and Neuroscience, University of Cambridge, Cambridge CB2 3EG, UK; 2 Department of Biochemistry, University of Cambridge, Cambridge CB2 1QW, UK

**Keywords:** channel blocking, Allotopic Model, Syntopic Model, 5-HT_3_A receptors, synergism

## Abstract

Drugs are often used in combination and, for pharmacologists, the manner of their interactions can cast light on drug mechanisms and biological processes. Here we provide simplified descriptions of commonly used analytical methods for analysing drug combinations and describe a new and practical experimental solution to address the mechanistic question: ‘Do two channel-blocking drugs bind at the same site?’ We define two simple mathematical models that describe the effects of two channel blockers acting simultaneously at either the same (Syntopic Model) or different (Allotopic Model) binding sites within a channel pore. We find that the optimum concentrations of two drugs for distinguishing between the two models are related to the mathematical Golden Ratio.

## Highlights

• A method to identify binding sites of ion channel-blocking drugs.
• The Golden Ratio in pharmacology.

## Analysing drug combinations

Drug combinations are commonly used in clinical practice to deliver therapeutic benefit beyond the use of single treatments alone [1–4]. Pharmacologists also use drug combinations to investigate drug mechanisms. For example, Schild analysis, using an agonist and antagonist, may reveal a competitive mechanism of action. However, such combinations do not always generate easily interpretable data. Much has been written on the analysis [5,6] and definition of responses as additive, synergistic or antagonistic (Box 1). Two related approaches are isobolograms (Box 2) and the Combination Index (CI) [7,8]. These require the estimation of pairs of drug concentrations that elicit equivalent responses. Such experiments are time-consuming, may require substantial quantities of drugs, and are conceptually operational rather than mechanistic.

Here we describe a simple method for addressing the question ‘Do two channel-blocking drugs bind at the same site?’ We define two mechanistically distinct models: a Syntopic Model with a single binding site for both drugs and an Allotopic Model with two distinct drug binding sites [9]. To distinguish between these models, concentrations of drug are combined that, when used alone, cause inhibition equal to the reciprocal of the Golden Ratio (≈61.8%) (Box 3) [10]. This approach may be particularly useful where the quantities of novel compounds are in limited supply or where there are no high quality reporter ligands.

## Theoretical modelling

Channel-blocking drugs are particularly well-suited for the mechanistic analysis of drug combinations, because inhibition of conductance is closely related to binding site occupancy. More specifically, our analysis is based on the following assumptions.

(i) Channel blockers reduce conductance to zero ‘much as a cork stoppers a bottle’ [11] and do not induce intermediate conductance states. It follows that at sufficiently high concentrations they will abolish the current response and at lower concentrations inhibition will be directly proportional to binding, as observed with saxitoxin [12–15]. Hence, zero occupancy results in zero inhibition (*In* = 0) and 100% occupancy results in complete inhibition (*In* = 1).
(ii) Channels function independently of each other, such that the activity of one channel is unaffected by the binding of a blocker to another.
(iii) Blocker binding is non-cooperative with a Hill coefficient of one. The mechanistic interpretation of non-unity Hill coefficients is not straightforward [16].
(iv) Blockers do not modify channel gating, for example, by changing the binding affinity or efficacy of a channel agonist.
(v) Blockers bind non-selectively to open and closed states.
(vi) Calculated block is assumed to be at dynamic equilibrium.

**Figure 1:**
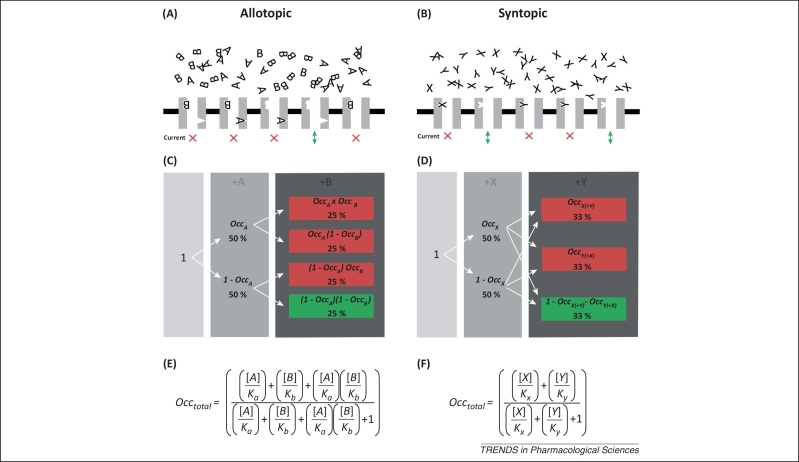
An illustration of channel occupancy. The Allotopic Model **(A)** describes the binding of two channel-blocking drugs (A and B) to separate and independent binding sites within a channel. The Syntopic Model **(B)** describes the binding of two channel-blocking drugs (X and Y) that share a common binding site where the binding of one blocker prevents binding of the other; it is a model of competitive binding. On application of one drug alone, channel occupancy may be modelled with the dissociation constant. On application of two drugs together, overall channel occupancy will depend on the dissociation constants and whether the drugs bind allotopically or syntopically. Only channels that have no drug bound pass current. **(C)** In the presence of both drugs together, the occupancy relationship between a binding site and its ligand remains unchanged in the Allotopic Model; occupancy by drug A is the same in the presence of drug A alone or drugs A and B. **(D)** In the Syntopic Model, occupancy by drug X differs depending on whether it is applied alone or in combination with drug Y. **(E,F)** Mathematical models relating overall occupancy to drug concentrations and dissociation constants. *K*_x_ = dissociation constant of drug X; *Occ*_X_ = proportion of channels occupied by drug X in the presence of X alone; *Occ*_X(+Y)_ = proportion of channels occupied by drug X in the presence of X and Y; *Occ*_*total*_ = proportion of channels occupied by any drug. (C) and (D) represent the situation when the concentration of each drug is equal to its dissociation constant.

Two distinct models for the simultaneous action of two channel-blocking drugs are defined (Figure 1).

### Allotopic Model

The Allotopic Model describes two drugs (A and B) that can bind to different sites at the same time (Figure 1A). The term ‘allotopic’ is used to indicate binding location [9] rather than ‘allosteric’, which implies conformational differences [17]. Here we assume that there is no allosteric modulation. The case where the affinity of one blocker is affected by the binding of the other is considered later.

Reversible interactions of drug A (and analogously for drug B) with an ion channel may be modelled with a simple rectangular hyperbola [18]:

[1] $$P_{\text{A}}=\frac{[A]/K_{\text{a}}}{[A]/K_{\text{a}}+1}\text{,}$$

where *P*_A_ is the proportion of binding sites occupied by drug A (0 ≤ *P*_A_ ≤ 1), [*A*] is the concentration of drug A, and *K*_a_ is the equilibrium dissociation constant of drug A.

Because drugs A and B bind independently to different sites, when used together the level of inhibition will be governed by laws of probability for independent, mutually non-exclusive events. The level of conductance will be equal to the product of the proportionate conductances in the presence of each drug alone. For example, if drug A alone reduces conductance by 30% (conductance = 0.70) and drug B reduces conductance by 40% (conductance = 0.60), conductance in the presence of the same concentrations of drugs A and B together would be 70% × 60% = 42% (inhibition = 58%) (Figure 1C). Therefore, the overall level of inhibition $\left(I{\text{n}}_{\text{A,B}}\right)$ would be:

[2] $$I{\text{n}}_{\text{A,B}}=1-\left[\left(1-I{\text{n}}_{\text{A}}\right)\left(1-I{\text{n}}_{\text{B}}\right)\right]\text{,}$$

which simplifies to:

[3] $$I{\text{n}}_{\text{A,B}}=I{\text{n}}_{\text{A}}+I{\text{n}}_{\text{B}}-I{\text{n}}_{\text{A}}I{\text{n}}_{\text{B}}\text{.}$$

### Syntopic Model

The Syntopic Model describes two drugs (X and Y) that share a binding site such that when one binds the other cannot (Figure 1B). This is defined by the familiar equation for competitive interactions [19,20]:

[4] $$P_{\text{X}}=\frac{[X]/K_{\text{x}}}{[X]/K_{\text{x}}+[Y]/K_{\text{y}}+1}\text{,}$$

where *P*_X_ is the proportion of binding sites occupied by drug X (0 ≤ *P*_X_ ≤ 1), [*X*] and [*Y*] are the concentrations of drugs X and Y, and *K*_x_ and *K*_y_ are the equilibrium dissociation constants for drugs X and Y, respectively. Total channel occupancy by drugs X and Y (*P*_X,Y_) is therefore:

[5] $$P_{\text{X,Y}}=\frac{[X]/K_{\text{x}}+[Y]/K_{\text{y}}}{[X]/K_{\text{x}}+[Y]/K_{\text{y}}+1}\text{.}$$

In the presence of drug X (and analogously for drug Y), the level of inhibition $\left(I{\text{n}}_{\text{X}}\right)$ may be derived from Equation 1 as follows:

[6] $$[X]/K_{\text{x}}=\frac{I{\text{n}}_{\text{X}}}{1-I{\text{n}}_{\text{X}}}\text{.}$$

Unlike the Allotopic Model, binding of drug X changes in the presence of drug Y (Figure 1D). However, Equation 6 can be substituted into Equation 5 to define inhibition in the presence of drugs X and Y together $\left(I{\text{n}}_{\text{X,Y}}\right)$, expressed in terms of inhibition in the presence of either drug X or Y.

[7] $$I{\text{n}}_{\text{X,Y}}=\frac{\left(\frac{I{\text{n}}_{\text{X}}}{1-I{\text{n}}_{\text{X}}}\right)+\left(\frac{I{\text{n}}_{\text{Y}}}{1-I{\text{n}}_{\text{Y}}}\right)}{\left(\frac{I{\text{n}}_{\text{X}}}{1-I{\text{n}}_{\text{X}}}\right)+\left(\frac{I{\text{n}}_{\text{Y}}}{1-I{\text{n}}_{\text{Y}}}\right)+1}\text{,}$$

which simplifies to:

[8] $$I{\text{n}}_{\text{X,Y}}=\frac{I{\text{n}}_{\text{X}}+I{\text{n}}_{\text{Y}}-2I{\text{n}}_{\text{X}}I{\text{n}}_{\text{Y}}}{1-I{\text{n}}_{\text{X}}I{\text{n}}_{\text{Y}}}\text{.}$$

Both the Allotopic and the Syntopic Models are represented mathematically in terms of drug concentration and dissociation constants in Figure 1E,F.

## Theoretical evaluation of the two models

**Figure 2:**
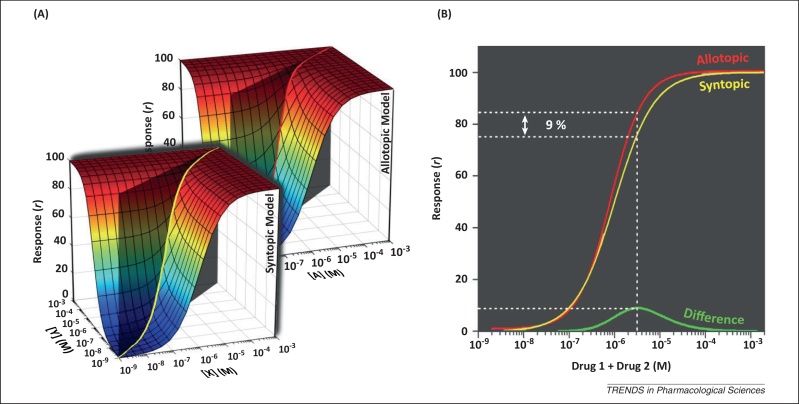
Comparison of Allotopic and Syntopic Models. The effects of two channel-blocking drugs acting **(A)** allotopically (drugs A and B) or syntopically (drugs X and Y). The difference between these two models is small and most clearly seen in a 2D view **(B)** of the response surface along the plane (grey shading in **A**) where [Drug 1] = [Drug 2]. The maximum difference between the models is approximately 9% and occurs when concentrations of drug equal to the dissociation constant multiplied by *ϕ* are used (Box 3). In these illustrations, the dissociation constants (*K*_d_) are 1 μM and the Hill coefficients are 1. Hence, when used alone, 1.618 μM of each drug causes inhibition of 61.8% and when used together the total drug concentration (3.24 μM) causes 85.4% inhibition in the Allotopic Model and 76.4% inhibition in the Syntopic Model (Box 3).

The predicted difference in inhibition caused by two drugs acting allotopically or syntopically is small and apparent only at certain concentration combinations (Figure 2). Therefore, drug concentrations must be carefully chosen to distinguish between the models. This difference $\left(I{\text{n}}_{DIFF}\right)$ is as follows:

[9] $$I{\text{n}}_{DIFF}=\frac{I{\text{n}}_{1}I{\text{n}}_{2}\left(1-I{\text{n}}_{1}\right)\left(1-I{\text{n}}_{2}\right)}{1-I{\text{n}}_{1}I{\text{n}}_{2}},\;(=[3]-[8])$$

where *I*n_1_ and *I*n_2_ are the levels of inhibition induced by the two drugs when acting alone.

The maximum difference between the two models occurs when the first partial derivatives of Equation 9 with respect to inhibition by each drug alone are equal to zero. Partial differentiation with respect to *I*n_1_ gives:

[10] $$\frac{\text{d}\left(I{\text{n}}_{DIFF}\right)}{\text{d}\left(I{\text{n}}_{1}\right)}=\frac{I{\text{n}}_{2}\left(1-I{\text{n}}_{2}\right)\left(I{\text{n}}_{1}^{2}I{\text{n}}_{2}-2I{\text{n}}_{1}+1\right)}{{\left(1-I{\text{n}}_{1}I{\text{n}}_{2}\right)}^{2}}\text{.}$$

An analogous expression may be derived for *I*n_2_. Solving Equation 10 for derivative = 0 gives two trivial solutions: *I*n_2_ = 0 and *I*n_2_ = 1. A more useful solution emerges from equating the third expression in the numerator to zero:

[11] $$I{\text{n}}_{1}^{2}I{\text{n}}_{2}-2I{\text{n}}_{1}+1=0\text{.}$$

Given the symmetry between *I*n_1_ and *I*n_2_, a solution may be found when *I*n_1_ = *I*n_2_:

[12] $$I{\text{n}}_{1}^{3}-2I{\text{n}}_{1}+1=\left(I{\text{n}}_{1}^{2}+I{\text{n}}_{1}-1\right)\left(I{\text{n}}_{1}-1\right)=0\text{.}$$

The solution to this expression is the reciprocal of the universally known Golden Ratio [10]: $I{\text{n}}_{1}=I{\text{n}}_{2}=\Phi =\left(\sqrt{5}-1\right)/2\approx 0.618$.

Therefore, to maximise the chance of observing a difference between the two models, concentrations of channel-blocking drugs should be used that cause 61.8% inhibition when acting alone. These concentrations are equal to IC_50_ × *ϕ*. At these concentrations, the predicted level of inhibition is ≈0.85 in the Allotopic Model and ≈0.76 in the Syntopic Model, giving a maximum possible *I*n_*DIFF*_ ≈ 0.09 (Box 3).

## Experimental design, statistical analysis and interpretation

To distinguish between allotopic and syntopic interactions, concentrations of two drugs are selected that cause ≈61.8% inhibition when used alone. The observed responses to these concentrations are then used to calculate predicted values for the Allotopic and Syntopic Models. These predicted values are compared with experimentally observed inhibition when both blockers are applied together.

Because the largest expected difference between the Allotopic and Syntopic Models is only 9%, its successful detection will depend on the variability of the data, and statistical analysis is necessary to compare the predicted and observed dual inhibition values. Deviations from the ideal level of inhibition by a single drug may be accommodated using a two-way analysis of variance (ANOVA) that treats this variability as a random effect, and the difference between the observed dual inhibition and the two predictions as a fixed effect. A significant overall ANOVA result is always expected because the allotopic and syntopic predictions are fundamentally different; therefore, non-significance is indicative of insufficient statistical power, which can be addressed by further experimental repeats. A *post hoc* Dunnett's test in which the observed dual inhibition is the reference value is suitable for evaluating the allotopic and syntopic predictions.

When the observed data differ significantly from one prediction but not from the other, this suggests that that the drugs act according to the latter model. If the observed inhibition is significantly different to both predictions, this suggests that the drugs bind allotopically, but with an allosteric effect.

## Experimental evaluation: the 5-HT_3_ receptor as a model system

**Figure 3:**
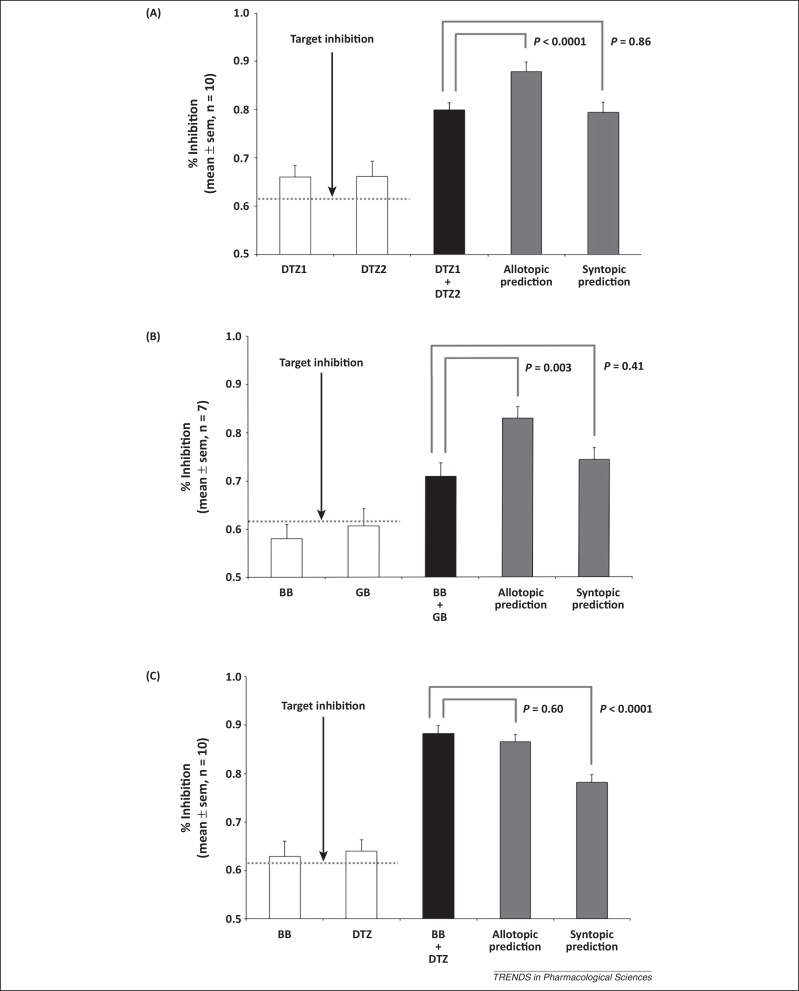
Inhibition of the 5-hydroxytryptamine type 3 (5-HT_3_) receptor by channel-blocking drugs. 5-HT_3_ receptors were activated with supra-maximal concentrations of 5-HT and inhibited with bilobalide (BB), ginkgolide B (GB) and diltiazem (DTZ) (each of which has a Hill coefficient of one [33], fulfilling assumption (iii) – see text) acting either alone or in combination. Concentrations of the drugs were selected to achieve inhibition of 61.8% when acting alone. Each panel shows observed data for the drugs acting alone (white bars) and for the same drugs acting together (black bars). The grey bars show the predicted levels of inhibition for the Allotopic and Syntopic Models, calculated using the experimental levels of inhibition caused by the drugs acting alone. Data are shown as the mean ± standard error of the mean (sem) and two-way analysis of variance (ANOVA) was used to determine whether there was a difference between the three sets of dual inhibition data. A *post hoc* Dunnett's test was used to compare the syntopic and allotopic predictions with the observed dual inhibition data. **(A)** Inhibition caused by two separate preparations of diltiazem (DTZ1 and DTZ2) matched the prediction made by the Syntopic Model but differed from the Allotopic Model, as expected for two preparations of the same drug. **(B)** Inhibition by BB and GB also closely matched the predicted Syntopic Model but differed from the Allotopic Model. Inhibition by a combination of BB and DTZ **(C)** was most closely predicted by the Allotopic Model and differed significantly from the Syntopic Model.

To test the utility of this method, we examined channel blockade of 5-hydroxytryptamine type 3 (5-HT_3_) receptors using bilobalide (BB), ginkgolide (GB) and diltiazem (DTZ) (Figure 3). Two separate preparations of the same drug would conform to the Syntopic Model, consistent with the model of Loewe (Box 1) [21]. As expected, the response to two separate concentrations of DTZ matched the Syntopic Model but differed significantly from the Allotopic Model (Figure 3A). Similarly, inhibition by BB and GB was consistent with published studies showing that these ligands share the same binding site (Figure 3B) [22]. By contrast, BB and DTZ, which have different channel binding sites [22], gave experimental values matching the Allotopic Model (Figure 3C).

## Relationship to other methods for analysing drug combinations

Much has been written on the analysis of drug combinations [5,23] and the challenge of defining drug synergy [3,5]. Here, we consider how our question: ‘Do two channel-blocking drugs bind at the same site?’ might have been addressed by other methods.

Isobolograms are often used to analyse drug combinations [8] and their patterns may reflect particular mechanisms of action (Boxes 1 and 2). Based on the concept of drug equivalence, this approach requires the identification of multiple pairs of drug concentrations that result in similar levels of response. This could be impractical where time and drug quantities are limited.

The Median Effect Plot [7] is identical to a Hill Plot and can therefore be used to determine Hill coefficients and IC_50_/EC_50_ (median effect) values for concentration–response relationships. Subsequent calculation of a Combination Index (CI) [24] uses equations that closely resemble those for isobolograms (Box 1): CI = 1 indicates additivity, CI < 1 synergy, and CI > 1 antagonism. Like individual isoboles, CI relates to a particular level of response. The analysis requires a median effect plot for each drug alone and for both drugs together in a fixed ratio and defines ‘mutually exclusive’ and ‘mutually non-exclusive interactions that resemble our Syntopic and Allotopic Models. However, there are analytical problems with this approach that have been described elsewhere [5,6].

Attempts have been made to interpret drug–drug interactions mechanistically [1]. However, drug combination analyses such as those described above have typically been used in clinical contexts to maximise therapeutic benefit and minimise adverse effects by combining drugs. They imply little about drug mechanisms [8]. Our approach is different, because it is explicitly and intentionally mechanistic.

## Concluding remarks

Compared with other methods, our approach requires minimal quantities of drug. Nevertheless, it offers insight into the binding sites of channel-blocking drugs by focussing on the point where the difference between the Allotopic and Syntopic Models is greatest. However, it would be naïve to suppose that these models adequately represent all possible situations where two channel-blocking drugs act simultaneously. Our Allotopic Model assumes that drugs bind independently, although interactions may occur. Allosteric effects induced by the binding of one drug may change the affinity of the other. This has been modelled using an affinity-modifying factor, often denoted as *α* [25,26]. A value of *α* = 0 is equivalent to our Syntopic Model and *α* = 1 to our Allotopic Model. Allosteric effects may be indicated when the observed dual inhibition differs from both syntopic and allotopic predictions, equivalent to *α* ≠ 1. Whether this effect is detectable will depend on its magnitude. At one extreme, the binding of one drug could allosterically prevent the binding of the other (i.e., *α* = 0) and this would be indistinguishable from the Syntopic Model, although similar outcomes would also arise from established methods for detecting competitive antagonism [25] such as Schild Analysis.

Future developments will incorporate the estimation of α in this simple approach. Preliminary modelling also indicates that, under appropriate conditions, our method can work for blockers that bind selectively to open channels and is not restricted to non-selective blockers. As a practical experimental method, non-labelled ligands may be used to reference binding sites, thereby eliminating the requirement for suitably labelled probes. Multiple pairwise comparisons would enable the mapping of channel binding sites, which may be useful for screening series of novel compounds, particularly those in scarce supply.

## Figures and Tables

**Figure I:**
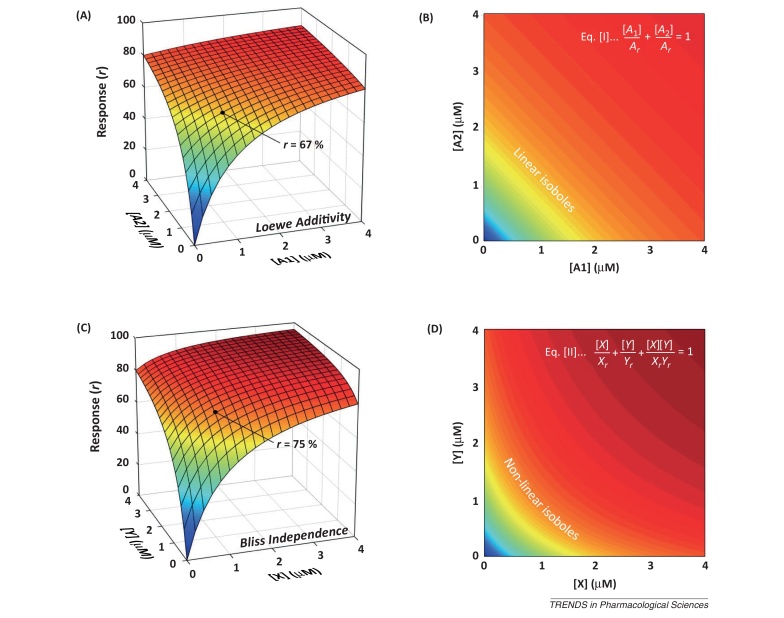
Different models to express drug interactions. **(A)** 3D plot illustrating Loewe Additivity as expected when the same drug (A) is applied on both horizontal axes. **(B)** Colour isobologram of Loewe Additivity showing linear isoboles. Equation I describes the isoboles for paired concentrations of drug A ([*A*_1_] and [*A*_2_]) that give a response *r* when used together. *A*_*r*_ is the concentration of A alone that elicits response *r*. Equation I also determines the Combination Index (CI) for mutually exclusive drugs. In this case, CI = 1. **(C)** 3D plot illustrating Bliss Independence. In Bliss’ original description, a combination of two LD_50_ concentrations of independently acting poisons results in a mortality of 75%. **(D)** Colour isobologram of Bliss Independence showing non-linear isoboles. Equation II describes the isoboles for paired concentrations of drugs X and Y that give a response *r* when used together. *X*_*r*_ and *Y*_*r*_ are the concentrations of X and Y alone that elicit response *r*. Equation II also determines the CI for mutually non-exclusive drugs. In this case, CI = 1.

**Figure I:**
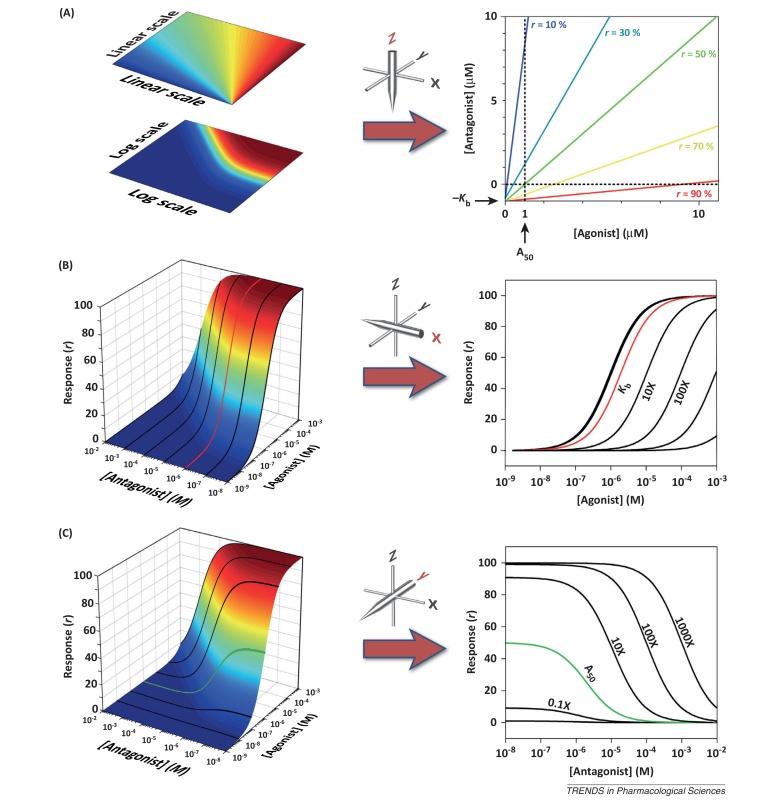
Three possible 2D views of a 3D surface representing the response to two drugs. **(A)** Viewed along the z-axis, the 3D plot reveals an isobologram in which the coloured contours represent equal response levels. **(B)** Viewed along the x-axis, the 3D plot of competitive antagonism appears as a Schild analysis in which agonist–response curves are contour lines representing equal antagonist concentrations. **(C)** Viewed along the y-axis, the 3D plot of competitive antagonism appears as a Cheng–Prusoff analysis in which antagonist–inhibition curves are contour lines representing equal agonist concentrations.

**Figure I:**
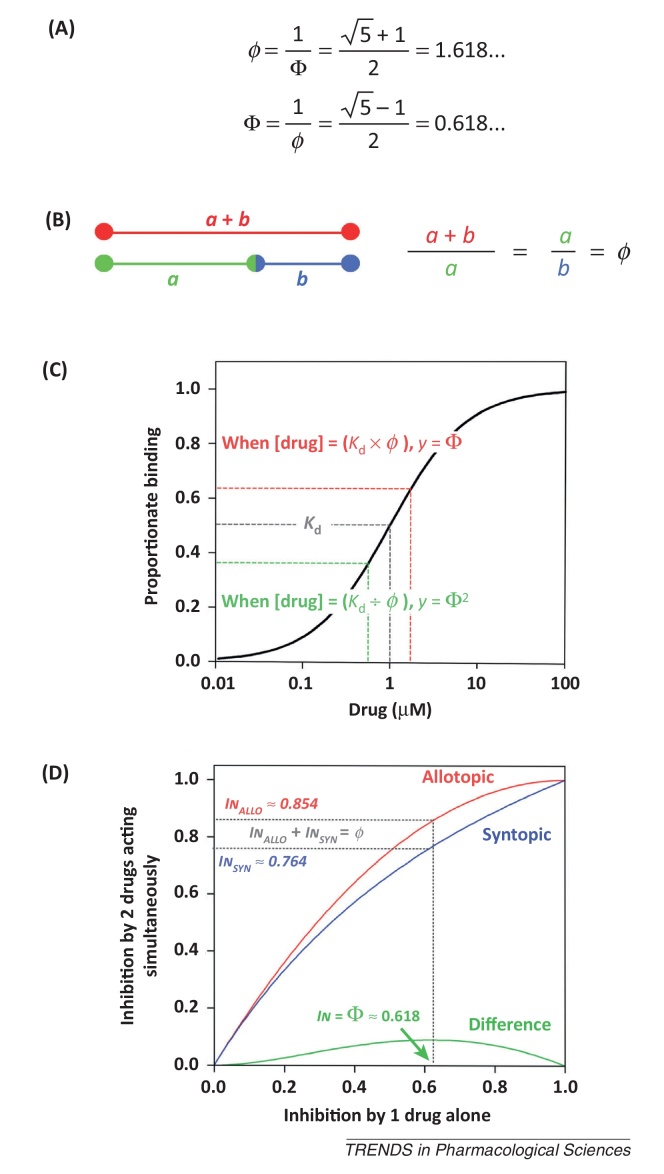
The Golden Ratio. **(A)** Arithmetical representation of the Golden Ratio (*ϕ*) and its reciprocal (Φ). **(B)** Euclid's geometrical representation of the Golden Ratio. **(C)** In a Hill–Langmuir concentration–response curve where the Hill coefficient = 1, the Golden Ratio is found at two locations either side of the central intersection between the dissociation constant (*K*_d_) and 50% binding. **(D)** The relationship between inhibition by channel-blocking drugs acting alone and as a pair, in the Allotopic and Syntopic Models. The maximum difference between the two models occurs when inhibition by a single drug is equal to Φ. At this point, the sum of allotopic and syntopic inhibitions is equal to *ϕ*.

## Box 1 When two drugs meet – additivity, synergy and antagonism

When used in combination with itself, a drug's response is determined by its own concentration–response relationship. This is Loewe Additivity [21] (Figure IA) and is analogous to our Syntopic Model; viewed as an isobologram (Figure IB), a linear pattern is revealed (Equation I). Bliss Independence is another model of drug combinations [27] (Figure IC) and is analogous to our Allotopic Model; in this case, the isobologram (Figure ID) reveals a non-linear pattern (Equation II). Both of these models may be considered additive (in both cases, the Combination Index [CI] = 1). When drug combinations result in greater responses than those predicted, synergy is indicated (CI < 1), whereas a response that is less indicates antagonism (CI > 1). However, for any drug pair it may be difficult to decide which of these models should be the reference for additivity. In the figure, for all drugs EC_50_ = 1 μM and Hill coefficient = 1.

## Box 2 What is an isobologram?

An isobologram (Figure IA) is one of three possible 2D views of a 3D surface representing the response to two drugs, in this case an agonist A and a competitive antagonist B. The other 2D views show Schild (Figure IB) [28] and Cheng–Prusoff (Figure IC) analyses [29]. Isobolograms are often shown with linear concentration scales and may have characteristic patterns. For competitive antagonism, the contours (or isoboles) are straight lines that converge where [*A*] = 0 and [*B*] = –dissociation constant (Figure IA). In the figure, A is the agonist: Maximum response = 100%, *pA*_50_ = 6, Hill coefficient = 1; and B is the competitive antagonist: *pK*_b_ = 6.

## Box 3 Phi-macology?

The Golden Ratio (Figure IA) has fascinated philosophers and scientists for centuries [10]. The name ‘phi’ (*ϕ*) is derived from the Greek sculptor Phidias who, it is claimed, made use of it in his work. *ϕ* has been associated with many natural phenomena, including nucleotide frequency in genomes [30], magnetic models of quantum physics [31], and plant phyllotaxis [32]. However, *ϕ* emerges most convincingly in mathematics and geometry. Around 300 BC, Euclid defined extreme and mean ratios using a segmented line (Figure IB). To our knowledge, no pharmacological association with *ϕ* has been described and yet it appears in the simplest of pharmacological models, the Hill–Langmuir equation (Figure IC) [18], as well as our Allotopic and Syntopic Models (Figure ID).

It is not obvious that *ϕ* should form part of the answer to the question: “Do two channel-blocking drugs bind at the same site?” Its unexpected appearance might invite speculation about further biological significance. However, whether simple pharmacological processes could provide a mechanistic basis for the emergence of *ϕ* in biological phenomena remains to be seen.
